# Macrophage Mediated Immunomodulation During *Cryptococcus* Pulmonary Infection

**DOI:** 10.3389/fcimb.2022.859049

**Published:** 2022-03-24

**Authors:** Yan Wang, Siddhi Pawar, Orchi Dutta, Keyi Wang, Amariliz Rivera, Chaoyang Xue

**Affiliations:** ^1^Public Health Research Institute, New Jersey Medical School, Rutgers University, Newark, NJ, United States; ^2^Department of Microbiology and Immunology , Guangdong Medical University, Dongguan, China; ^3^Center for Immunity and Inflammation, New Jersey Medical School, Rutgers University, Newark, NJ, United States

**Keywords:** *Cryptococcus neoformans*, macrophage, host immune response, cryptococcal evasion strategies, *C.neoformans*-macrophage interaction

## Abstract

Macrophages are key cellular components of innate immunity, acting as the first line of defense against pathogens to modulate homeostatic and inflammatory responses. They help clear pathogens and shape the T-cell response through the production of cytokines and chemokines. The facultative intracellular fungal pathogen *Cryptococcus neoformans* has developed a unique ability to interact with and manipulate host macrophages. These interactions dictate how *Cryptococcus* infection can remain latent or how dissemination within the host is achieved. In addition, differences in the activities of macrophages have been correlated with differential susceptibilities of hosts to *Cryptococcus* infection, highlighting the importance of macrophages in determining disease outcomes. There is now abundant information on the interaction between *Cryptococcus* and macrophages. In this review we discuss recent advances regarding macrophage origin, polarization, activation, and effector functions during *Cryptococcus* infection. The importance of these strategies in pathogenesis and the potential of immunotherapy for cryptococcosis treatment is also discussed.

## Introduction

Invasive fungal infections are a significant global health problem that affect diverse populations and where individuals with a weakened immune system are at high risk. *Cryptococcus species* complex, mainly *C. neoformans* and *C. gattii*, are fungal pathogens that infect humans *via* the respiratory tract to cause pulmonary cryptococcosis. *Cryptococcus* can often disseminate to infect other organ systems, including the central nervous system (CNS) where it can cause deadly cryptococcal meningitis. Cryptococcal meningitis accounts for roughly 181,000 deaths in AIDS patients, which is roughly 15% HIV/AIDS of all AIDS-related deaths annually (Leopold Wager et al., 2018). Despite medical advances, invasive fungal infections like cryptococcosis remain understudied and underdiagnosed, creating a significant public health burden. The incidence of cryptococcal infections has risen globally due to increased populations with weakened immunity. Predisposing factors for cryptococcosis include HIV, autoimmune diseases, prolonged use of immunosuppressants, diabetes, and organ transplantation, etc. Cryptococcosis is known as AIDS-defining illness in 60-70% of HIV-infected individuals (Mirza et al., 2003), resulting in ~15% of all AIDS-related deaths annually (Leopold Wager et al., 2018). The cryptococcal polysaccharide capsular antigen (CrAg) is an early marker of cryptococcal disease. CrAg can be detected in the serum approximately 3 weeks before the onset of symptoms, and is highly predictive of incident cryptococcal meningitis (Maziarz and Perfect, 2016). In addition, baseline titers of CrAg in serum and cerebral spinal fluid (CSF) correlate with fungal burden and prognosis in patients with cryptococcal meningitis (Kabanda et al., 2014). Current antifungal regimes commonly involve combination therapy of amphotericin B, flucytosine, and fluconazole (Perfect et al., 2010). Despite clinical intervention, the 3-month mortality of HIV patients with acute cryptococcal meningoencephalitis remains as high as 20% (Voelz and May, 2010). This poor prognosis has emphasized the need to explore alternative treatments, such as immunotherapy, passive immunization, and cytokine-based treatment strategies.

*Cryptococcus* infection begins with the inhalation of fungal spores or desiccated small yeasts from the environment into the deep alveoli of the lungs and cause pneumonia. Macrophages play an integral role in anti-cryptococcal defense. In particular, alveolar macrophages (AMs) act as first responders, where they detect and engulf cryptococcal cells (Giles et al., 2009). Additionally, opsonins detect the fungal spores by binding to distinct pattern-recognition receptors (PRRs) (Romani, 2004). In case of encapsulated yeast such as *C. neoformans*, absence of opsonin fails to mount efficient anti-fungal response (Levitz and Tabuni, 1991; Kelly Ryan et al., 2005). As facultative intracellular pathogens, *Cryptococci* are capable of survival and replication within host macrophages. Failure to clear pulmonary infection leads to fungal dissemination throughout the body and towards the brain, resulting in cryptococcal meningitis (Sabiiti et al., 2014). Both the innate and adaptive immune responses play significant roles in protection against cryptococcus. Hosts with intact immune system mount an immune response that leads to clearance of the fungus, or the establishment of a latent, asymptomatic infection accompanied by the formation of cryptococcoma. However, patients with impaired cell-mediated immunity are unable to effectively clear *C. neoformans*. Thus, effective innate immune activation and sufficient inflammatory responses are key to controlling cryptococcosis.

The complement system is an early defense against *Cryptococcus* in the bloodstream, performing the important function of preparing the host for subsequent responses. The two major functions of the complement system are to stimulate the chemotaxis of phagocytic effector cells and enhance their uptake of cryptococcal cells *via* opsonization. Engulfment of cryptococcal cells by a variety of innate effector cells has been demonstrated both *in vitro* and *ex vivo* in multiple studies. Innate effector cell engulfment examples have been demonstrated in rodent peritoneal and pulmonary macrophages, human neutrophils and macrophages, and swine microglia (Voelz and May, 2010). Phagocytosis is triggered by direct recognition of the yeast or by receptor-mediated recognition *via* complement or antibodies. Oxidative burst exerted by neutrophils has been demonstrated *in vitro* to effectively kill *C. neoformans* (Mambula et al., 2000).

Development of an adaptive immune response is also essential for overcoming *Cryptococcus* infection, including antibody and cell-mediated immune responses. Passive administration of capsule-binding antibody can prolong host survival and/or reduce fungal burdens in experimental cryptococcosis (Voelz and May, 2010). Meanwhile, passive immunization with two different monoclonal IgM antibodies against melanin can reduce fungal burden during mouse infection and is able to directly reduce cryptococcal growth *in vitro* (Rosas et al., 2001). Antibody and complement are required for efficient phagocytosis of *C. neoformans in vitro*, with potential compensation when one opsonin is missing or impaired. Furthermore, a B cell deficient murine model showed decreased AM uptake through decreased IgM resulting in increased fungal burden (Szymczak et al., 2013). This model suggests an association between B cell responses and phagocyte effector functions. Taken together, these reports suggest antibody-mediated phagocytosis likely plays an important role in the clearance of *Cryptococci*.

Evidence from mouse models suggests the protective effects of antibodies are at least partly due to interaction with cell-mediated immunity. During cryptococcal infections, dendritic cells (DCs) are regarded as the major initiators of protective cell-mediated immunity (Patel et al., 2017) and also function as antigen presenting cells (APCs) (Rossi and Young, 2005). DCs require opsonization to initiate anti-cryptococcal activity. Absence of opsonization fails to secrete detectable levels of IL-10 or IL-12 (Kelly Ryan et al., 2005). However, compared to AMs, DCs are more potent inducers of T-cell activation *in vivo*. Furthermore, major cryptococcal antigens (e.g. mannoproteins and glycoantigens) are predominantly presented to T-cells by DCs (Voelz and May, 2010).

Several types of T-cells are involved in host responses to *C. neoformans*, including CD4^+^, CD8^+^ and natural killer (NK) T cells. All these T-cells exhibit direct antimicrobial activity to *C. neoformans*, and T cell secreted proteins such as granulysin and perforin can induce both cryptococcal permeabilization and lysis (Ernst et al., 2000; Voskoboinik et al., 2006; Zheng et al., 2007). CD4^+^ T-cells are key for orchestrating responses from various arms of the adaptive immune system. Once activated, CD4^+^ T-cells differentiate into different specific subsets based on the local cytokine milieu. Th1 and Th17 cytokines activate, whereas Th2 cytokines inhibit anti-cryptococcal functions. Their recruitment and secretion of cytokines such as TNF-α and IFN-γ or TGF-β and IL-6, results in protective anti-fungal responses. In a study with J774 macrophage cell line, Th1 and Th17 stimulated macrophages significantly increased the phagocytosis of *Cryptococci*, controlled the cryptococcal intracellular proliferation than Th2-stimulated cells (Voelz et al., 2009). As proof of concept, a murine model of cryptococcosis also demonstrated the role Th1 and Th17 cells in host defense (Muller et al., 2007; Zhang et al., 2009; Wozniak et al., 2011). Conversely, stimulation with IL-4 and IL-13 leads to Th 2 immunity, which is characteristically non-protective during fungal infections (Blackstock and Murphy, 2004; Milam et al., 2007; Muller et al., 2007; Wormley et al., 2007). In AIDS patients with cryptococcal meningitis, presence of IFN-γ/TNF-α-producing peripheral CD4+ T cell response is associated with increased concentration of IL-10 and IL-17, correlates with beneficial clinical outcomes in patients (Jarvis et al., 2013, Mora et al., 2015). Moreover, protection induced by the most of cryptococcus vaccine candidates are associated with early increase in pulmonary T cell infiltrates and the induction of a strong Th1 and Th17 responses (Chaturvedi et al., 2014; Specht et al., 2015; Zhai et al., 2015; Upadhya et al., 2016; Masso-Silva et al., 2018; Wang et al., 2019). Another dendritic cell-based vaccine against *C. gattii* demonstrated lung-resident memory Th17 cells produced IL-17A which in turn, suppressed lung fungal burden and improved the survival of mice (Ueno et al., 2019). Thus, the Th1-Th2-Th17 balance is essential for protection against *Cryptococci*.

Macrophages are thought to be the primary effector cells for killing and ultimately clearing a cryptococcal infection. Following phagocytosis, AMs not only directly kill the invading microorganisms, but also initiate and modulate appropriate downstream immune responses. These responses include the release of cytokines, presentation of antigen, as well as activation and recruitment of other immune cells. Evidence from clinical findings, animal models, and *in vitro* experiments point the importance of macrophages. Immune defects in macrophage development, activation, proliferation, and signaling all lead to increased risk of disseminated cryptococcal infection (Leopold Wager et al., 2014; Leopold Wager et al., 2015; Campuzano et al., 2020; Viola et al., 2021). However, despite this known functional importance, the details of how macrophages function during clearance of cryptococcal infections remains incomplete. Here, we summarize general macrophage functions and the impact of macrophage-cryptococcus interaction on the outcome of infection. If the goal of immunotherapy as an adjunct to antifungal treatment is to be implemented, it is critical to understand the precise effector pathways and mechanisms that are required.

## Macrophage Development and Antimicrobial Function

### Macrophage Development

Tissue-resident macrophages are derived from either blood monocytes or through local proliferation of yolk sac-derived phagocytes that are originally seeded during embryonic development (McGrath et al., 2015; Das Gupta et al., 2016). Depending on the organ they populate, macrophages receive different designations. Tissue-specific macrophages are distinguished by specific differentiation programs, cell morphologies, and specialized functions (Jurberg et al., 2018). Examples of such macrophages include microglia in the brain, alveolar macrophages in the lungs, Kupffer cells in the liver, osteoclasts in the bone, and chondroclasts in the cartilage. Self-renewal is the defining hallmark of most tissue-resident macrophages.

**Figure 1:**
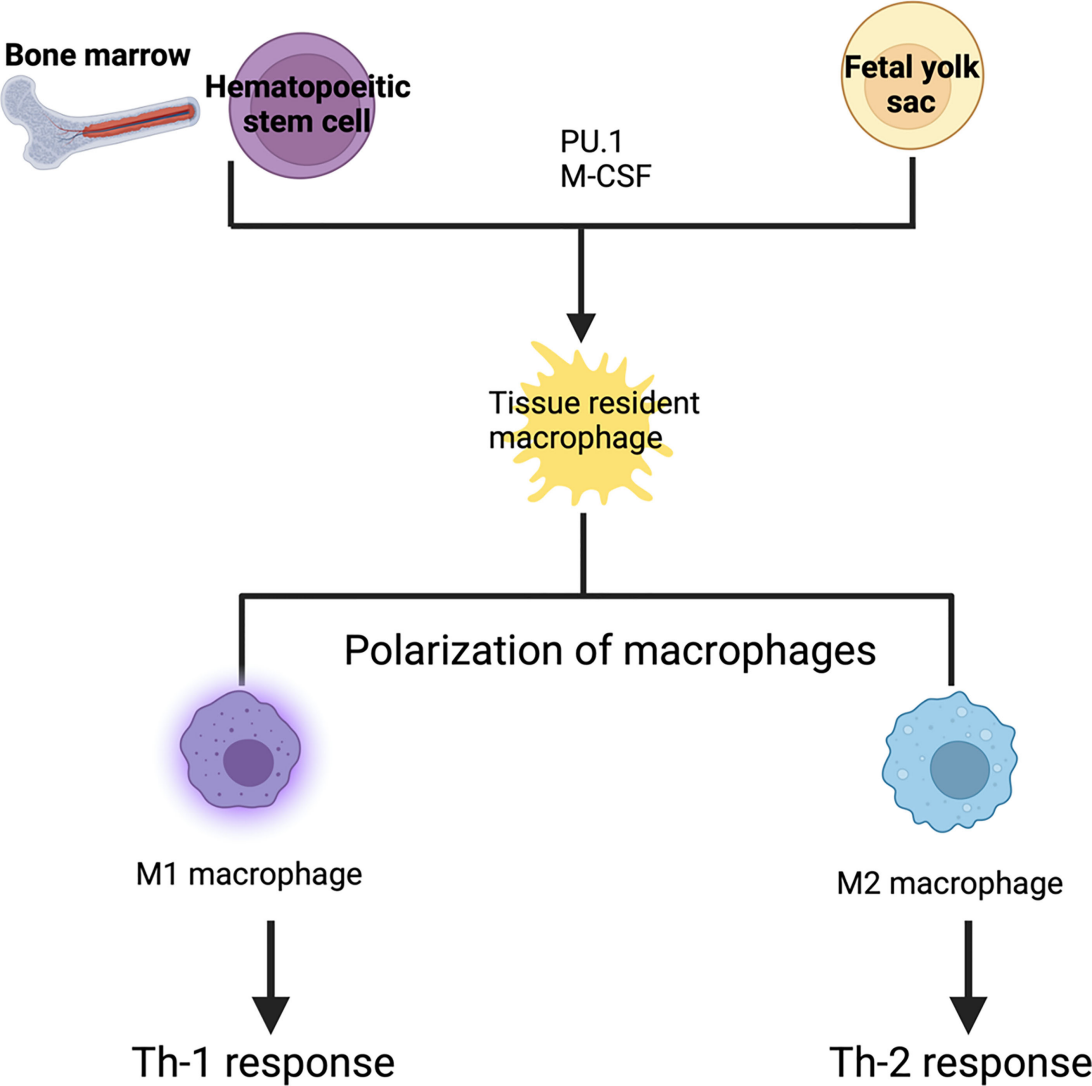
Development of tissue resident macrophages: Tissue resident macrophages are derived from either blood monocytes or fetal yolk sac. Depending upon the signals they receive, they form tissue specific macrophages. PU.1 and macrophage colony stimulating factor are required for the differentiation into tissue resident macrophages. These tissue resident macrophages can be polarized into either M1 or M2 macrophages. M1 macrophages are activated macrophages which result in a Th-1 response (killing intracellular pathogen) whereas M2 macrophage are alternatively activated macrophages which result in a Th-2 response (wound healing, and tissue repair).

Macrophage development is dependent on the transcription factor PU.1 and macrophage colony-stimulating factor receptor (CSF1R) signaling (Kurotaki et al., 2017). Transcription factor PU.1 (encoded by the Spi1 gene) is a master switch that controls monocyte/macrophage development from hematopoietic stem cells (Roy, 2016). Nearly all macrophages, monocytes and mature B cells are absent in Spi1^–/–^ mice (Scott et al., 1994; McKercher et al., 1996). CSF1R signaling is required for the differentiation, survival and proliferation of macrophages and monocytes. Indeed, CSF1R-deficient mice show severely reduced numbers of most tissue-resident macrophages and monocytes (Dai et al., 2002). CSF1 and interleukin 34 (IL-34) are the two known ligands for CSFR1. CSF1 is abundant in many tissues including the lymph nodes, uterus, ovary, salivary gland and peripheral blood, whereas IL-34 is specifically expressed in the epidermis and brain (Wang and Colonna, 2014). Binding of these cytokines to CSF1R activates the PI3K–Akt and Ras–Raf–MEK–ERK signaling pathways, thereby controlling various downstream cellular processes including cytoskeletal remodeling, cell adhesion, survival, and proliferation (Pixley and Stanley, 2004; Mouchemore and Pixley, 2012). Blockade of CSF1R signaling suppresses the proliferation of tissue resident macrophages *in vivo* during inflammation (Davies et al., 2013). Thus, CSF1 and IL-34 are potent trophic factors for macrophages and monocytes (**Figure 1**).

### Macrophage Receptor

Macrophages, as key innate immunity cells, express a plethora of receptors that allow them to sense the extracellular environment with exquisite sensitivity and specificity. Examples of these receptors include phagocytic receptors, cytokine receptors, receptors for other host-derived inflammatory mediators and several families of pattern recognition receptors (PRRs), such as the Toll-like receptors (TLRs) and Nod-like receptors (NLRs) (Das Gupta et al., 2016). Macrophages activated through PRRs produce potent pro-inflammatory cytokines, such as TNF-α, IL-1β, IL-6, and IL-12, together with chemokines and toxic free radicals (Arango Duque and Descoteaux, 2014; Kawasaki and Kawai, 2014; Turner et al., 2014). Scavenger receptors bind a diverse range of ligands from bacteria to native proteins, allowing them to regulate both cell adhesion and the removal of noxious agents by phagocytosis (Prabhudas et al., 2014). Recognition and clearance of apoptotic cells is another important function of macrophages. Apoptotic cells are detected by macrophages through specific receptors that recognize externalized phosphatidylserine (PS) on the cell membrane of dying cells (Jurberg et al., 2018). Macrophages are also able to detect complement molecules by using cognate receptors and antibodies by using Fc receptors. These molecules opsonize pathogens and abnormal cells, thus stimulating their phagocytosis. The combined actions of these detection systems enable macrophages to initiate appropriate inflammatory programs upon perturbed homeostasis.

### Macrophage Activation and Polarization

Macrophages are dynamic cells and can undergo transitions across a continuum of phenotypes and activation states. Depending on the microenvironmental milieu, macrophages are polarized into one of two subclasses, broadly classified as M1 or M2 (Mantovani et al., 2002; Mosser and Edwards, 2008; Murray et al., 2014). M1 macrophages, also known as “classically activated,” are induced by the canonical Th1 cytokines TNF-α and IFN-γ. In addition, granulocyte–macrophage colony-stimulating factor (GM-CSF), lipopolysaccharide (LPS), and other TLR ligands also stimulate M1 polarization. In contrast, polarization of M2, or “alternatively activated macrophages (AAMs),” is induced by Th2 cytokines, IL-4 and IL-13 (**Figure 1**). The signal transducer and activator of transcription 1 (STAT1) and interferon-regulatory factor 5 (IRF5) play a major role in dictating M1 macrophage phenotypes, whereas, STAT3/STAT6, peroxisome proliferator-activated receptor-γ (PPARγ), and IRF4 direct M2 macrophage polarization (Lawrence and Natoli, 2011). M1 macrophages support Th1 responses and produce large amounts of reactive oxygen species (ROS) and nitric oxide (NO) to kill intracellular pathogens (Mantovani et al., 2002). Meanwhile, M2 macrophages are involved in tissue repair, vascularization, tumor promotion/invasion, and in responding to parasitic infections. AAMs are also characterized by their high expression of scavenger receptors in addition to high production of IL-10, VEGF, and MMPs (Mantovani et al., 2002). In cryptococcosis, STAT1-mediated M1 macrophage activation is critical to generate a host protective effector Th1 cell population (Leopold Wager et al., 2014; Leopold Wager et al., 2015).

Macrophages play diverse roles. Resident macrophages regulate tissue homeostasis by acting as sentinels and respond to changes in physiology as well as external challenges. Macrophages are uniquely equipped to sense and respond to tissue invasion by infectious microorganisms and tissue injury through various scavenger, pattern recognition and phagocytic receptors (Lavin et al., 2015). Macrophages also have homeostatic functions, such as the clearance of lipoproteins, debris and dead cells using sophisticated phagocytic mechanisms (Kohyama et al., 2009; Hussell and Bell, 2014). Unfortunately, these homeostatic and reparative functions are sometimes subverted due to continuous insult, resulting in causal association of macrophages with disease states, such as the inflammatory diseases of fibrosis, obesity, and cancer. Thus, macrophages are an incredibly diverse set of cells constantly shifting their functional state to new set points in response to changes in tissue physiology or environmental challenges.

## Macrophage Mediated Host Defense Against *Cryptococcus* Infection

Macrophages represent the first line of defense against invading microbial pathogens. For example, AM can survey inhaled pathogens on the pulmonary surface (Neupane et al., 2020). Macrophages recognize bacteria *via* pathogen-associated molecular patterns (PAMPs), vesicular, or cytoplasmic PRRs. Downstream signaling cascades linked to these receptors induce receptor-mediated phagocytosis, a hallmark of host defense. In addition, pathogen recognition also triggers a variety of pro-inflammatory responses. These functions are driven by expression of chemokines and cytokines, as well as through the secretion of anti-microbial effectors. Understanding the interactions between lung macrophages and *Cryptococci* is the key to understand fungal pathogenesis and local inflammatory responses. The outcome of the *C. neoformans*-macrophage interaction can predict infection outcomes, however, the molecular mechanisms of these interactions remain incompletely understood (Dragotakes et al., 2019).

Upon infection, resident macrophages and dendritic cells in the lungs mediate initial immune response by phagocytosis of *C. neoformans* (Rudman et al., 2019). Phagocytosis is regulated by a diverse set of factors, including antibodies, complement proteins, surfactant protein D or the scavenger receptors SCARF1 and CD36 (Zhang et al., 2015). Fcγ receptors (FcγRs) on macrophages can bind and mediate phagocytosis of antibody-opsonized yeast cells. Furthermore, interaction of IgG1 complexes with related FcγRs facilitates either fungal killing, fungal growth inhibition through macrophage-mediated antibody-dependent cytotoxicity, or macrophage phagocytosis. IgM and IgA specific to the major capsular component - glucuronoxylomannan (GXM) promote complement-independent and CD18-dependent phagocytosis (Zhang et al., 2015). Phagocytosis of *C. neoformans* by lung macrophages is significantly impaired in IgM deficient mice (Subramaniam et al., 2010). In contrast, IgG3-mediated phagocytosis is associated with structurally different FcγR (Saylor et al., 2010).

Host cytokine factors also influence expulsion or proliferation of *C. neoformans* by changing the composition of the phagosome. Th1 and Th17 cytokines decrease non-lytic exocytosis and are more efficient at containing *Cryptococci*. Conversely, Th2 cytokines augment the extrusion of *C. neoformans* out of macrophages, which may contribute to the extravasation of *C. neoformans* and aggravated disease (Zhang et al., 2015). Several studies have shown that macrophage polarization status is a key factor in host responses against cryptococcal infection. Activation of M1 macrophages leads to optimal fungicidal effects. M1 activity is associated with robust production of reactive oxygen and nitrogen species to establish control of the *C. neoformans* infection (Basso et al., 2020). Conversely, M2 macrophage promotes a Th2-derived interleukin IL-4/IL-13-dominant cytokine environment, resulting in uncontrolled fungal growth, dissemination, and exacerbation of disease (Basso et al., 2020). In summary, M1 macrophages are the major cells associated with fungal clearance, whereas M2 macrophages serve as intracellular reservoirs of *C. neoformans*. Similarly, polarized cytokine environments are the most influential determinants of macrophage activation phenotypes. Proliferation of *C. neoformans* within macrophages is also significantly lower following treatment with IFN-γ and IL-17 compared with IL-4 and IL-13 (Hardison et al., 2012).

Pulmonary H99 infection in BALB/c mice is typically accompanied by nonprotective Th2-type cytokine responses and M2 activation. This induces type 2 responses that are characterized by increased IL-4 and IL-13 cytokine production, M2 macrophage polarization, increased fungal burden, dissemination, and exacerbation of disease (Leopold Wager et al., 2015). Experimental pulmonary *C. neoformans* infection in IFN-γ knockout (KO) mice significantly increased macrophage recruitment as compared to wild-type mice (Arora et al., 2005). However, *C. neoformans* infected IFN-γ KO mice also displayed type 2 polarized responses, resulting in M2 differentiation and reduced fungi stasis with progressive cryptococcal infection. Indeed, induction of M1 macrophages in an IL-4/IL-13KO mouse model of pulmonary cryptococcosis was associated with improved control of fungal burden and overall improved lung pathology. However, these animals ultimately succumbed to infection, suggesting that the absence of type 2 cytokines alone is not sufficient to confer prolonged protection (Zhang et al., 2009). In another model of pulmonary cryptococcosis, mice were infected with a strain of H99 engineered to express IFN-γ, designated H99γ, for driving activation of M1 macrophages. Within these mice, there was increased Th1-type and IL-17A cytokine responses and subsequent resolution of the acute infection (Leopold Wager et al., 2015). Critically, mice that were protectively immunized with the *C. neoformans* strain H99γ and then subsequently challenged with H99 developed an M1 macrophage phenotype that was associated with enhanced STAT1 activation and strong protection against the yeast (Hardison et al., 2012). Further clarification of the role played by STAT1 and its downstream effectors in facilitating protection against *C. neoformans*, and other intracellular organisms has the potential to identify novel strategies for immune therapies that target host responses, rather than the invading organism. Taken together, these studies suggest efficient control of *C. neoformans* infection occurs following activation of M1 macrophages and downstream Th1 responses. In contrast, M2 activation is associated with Th2 responses and favors *C. neoformans* growth and the establishment of a latent infection (De Leon-Rodriguez et al., 2018b).

## Mechanisms Underlying *Cryptococcus* Evasion of Macrophage Defense

*C. neoformans* thrives on a battery of virulence factors, including polysaccharide capsule, melanin, fungal proteins, titanization, and novel mechanisms to evade phagocytosis.

### Capsule

**Figure 2:**
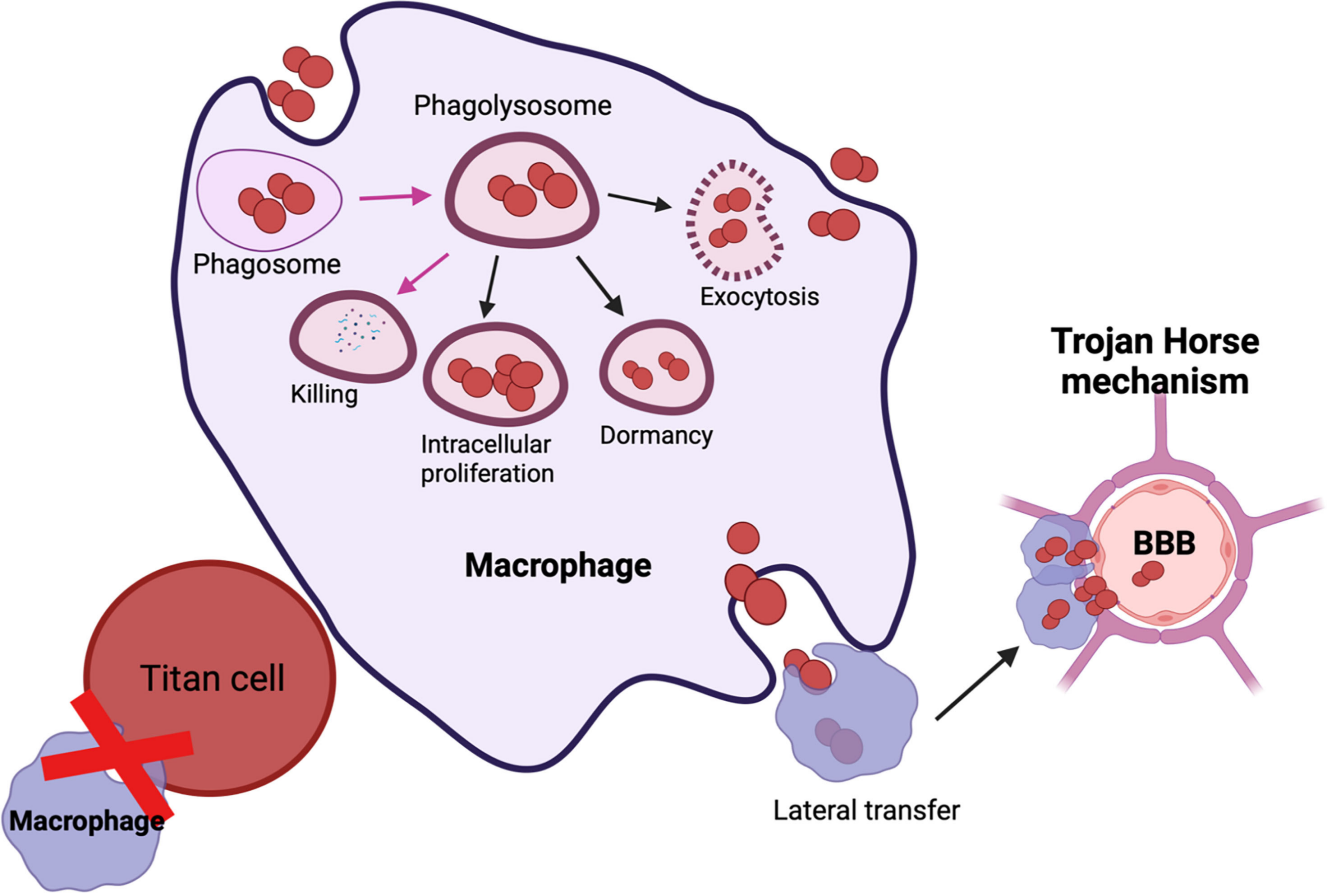
Possible outcomes of *C. neoformans*-macrophage interaction: Engulfment of *C. neoformans* results in containment within the phagolysosome and *C. neoformans* killing (shown in pink arrows). *C. neoformans* has multiple methods of interacting with host immune responses which include: (i) Intra-cellular proliferation, (ii) Dormant/latent, (iv) Titanization or exiting the macrophage *via* **(A)** exocytosis or **(B)** lateral transfer. Lateral transfer can also contribute to the dissemination of *C. neoformans* to the brain *via* Trojan horse mechanism.

*C. neoformans* is a fungal pathogen with a thick polysaccharide capsule. This capsule plays the dual role of “sword” by evading host immune responses and also acts as “shield” by protecting the fungi from the host’s anti-microbial oxidative burst attacks and phagocytosis. Interaction of *C. neoformans* with macrophages either results in (a) lysis of host cells due to capsular enlargement, (b) lysis of the fungi or (c) survival of both the host and the pathogen (**Figure 2**). In mammalian hosts, all possible outcomes result in either survival of host, active infection, or emergence of dormant/latent infection. The polysaccharide capsule is composed of three major components namely: glucuronoxylomannan (GXM), galactoxylomann (GalXM) and mannoproteins (MP). GXM influences the function of innate immune cells like macrophages, neutrophils, and dendritic cells by reducing the antigen presentation functions, altering costimulatory molecules, dysregulating secretion of proinflammatory and anti-inflammatory cytokines and by stimulating apoptosis *via* upregulation of the death receptor Fas/FasL. Overall, GXM has immunosuppressive effects on innate immune cells. Similarly, immunosuppressive effects of GalXM also inhibit adaptive immune responses by inducing T cell apoptosis and suppressing B cell activity (Decote-Ricardo et al., 2019) In addition to the immunosuppressive activity of the capsule, chemically diverse signals in the host, such as iron deprivation, serum, CO_2_, can induce capsular enlargement to evade phagocytosis. Conversely, MPs stimulate a protective cell-mediated immune response against *C. neoformans* (Murphy, 1998; Levitz and Specht, 2006). MP1 and MP2 bind to dendritic cell mannose receptors to stimulate efficient antigen presentation, secrete pro-inflammatory cytokines (IL-12, IFN-γ, and TNF-α), and induce T cell proliferation (Pietrella et al., 2005). Furthermore, mannoprotein antigen MP98 and MP88 is known to stimulate T cell responses (Levitz et al., 2001; Huang et al., 2002; Dan et al., 2008). Another study characterized two novel MPs-MP 84 and MP115 which are recognized by serum antibodies during cryptococcosis (Biondo et al., 2005; Teixeira et al., 2014).These immunological features make MPs a potential vaccine candidate. Overall, the capsule exerts immunosuppressive effects on the host.

### Melanin

Laccase enzymes catalyze the synthesis of melanin. Therefore, laccase deficient fungal strains also show defective melanin production (Kwon-Chung et al., 1982, Rhodes et al., 1982). Melanin has antioxidant activity (Wang and Casadevall, 1994a; Wang and Casadevall, 1994b), mediates resistance to antifungal drugs (van Duin et al., 2002; Wozniak and Levitz, 2008) such as amphotericin B and caspofungin and provides rigidity to the cell wall. Melanin can also be recognized by host antibodies and drive a protective immune response (Rosas et al., 2001). *C. neoformans* has two laccase (Lac) isoforms, Lac1 and Lac2. Lac1 predominantly plays a role in infection and is expressed in the cell wall, whereas Lac2 is expressed in the cytoplasm (Polacheck et al., 1982; Missall et al., 2006). Furthermore, the investigators utilized a congenic pair of laccase positive and laccase deficient strains to determine the role of antifungal activity in murine AM. Using the recombinant cryptococcal laccase, they found oxidation of phagosomal iron with a resultant decrease in hydroxyl radical formation protects *C. neoformans* (Liu et al., 1999). Moreover, laccase also has immunomodulatory effects by catalyzing prostaglandin E2 production, which helps sustain infections (Erb-Downward and Huffnagle, 2007). Furthermore, laccase contributes to cryptococcal CNS dissemination *via* increased eosinophilia, M2 macrophage response, overall polarizing CD4+T cells towards a Th2 immune response (Qiu et al., 2012). Another study associated laccase activity with cryptococcal survival in human fluid (CSF) *ex-vivo* and poor fungal clearance (Sabiiti et al., 2014). Laccase also modulates nonlytic exocytosis, a function independent of melanin production. *C. neoformans* isolates that produce melanin faster frequently undergo nonlytic exocytosis resulting in increased dissemination of fungal cells (Frazão et al., 2020).Overall, the dual actions of melanin and laccase synergize for full virulence and promote dissemination of yeast cells.

### Titan Cell

*C. neoformans* cells show great heterogenicity in cell size during lung infection. *C. neoformans* possess a rare and complex ability to dramatically enlarge their size from a typical 5 μm cell body diameter up to 100 μm, thereby forming “titan cells”. Recent studies revealed that such morphologic alterations play an important role in fungal adhesion and penetration of biologic membranes for avoiding immune detection, and promoting fungal dissemination (Okagaki et al., 2010; Zaragoza et al., 2010; García-Barbazán et al., 2016). Thus, titan cells play a key role in pathogenesis due to their ability to survive and disseminate within the host.

Importantly, the main virulence factor of *C. neoformans*, capsule, has structural differences in titan cells, including being more dense, having an abnormally thick cell wall and having more crosslinked capsule (Zaragoza et al., 2010). The significantly enlarged cell size also corresponds to increased DNA content and undergoes endoreduplication to form polyploid cells containing 16, 34, 64, or more copies of the genome (Okagaki et al., 2010; Zaragoza et al., 2010; Kronstad et al., 2011). Despite polyploidy, titan cells produce haploid progeny, highlighting a distinct developmental transition likely required to disseminate into the CNS. Interestingly, like titan cells, their haploid progeny is also resistant to stress factors and antifungals (Zaragoza et al., 2010; Gerstein et al., 2015).

Recent descriptions of the *in vitro* conditions required to promote titanization have contributed to a better understanding of these cells. Titan cells can be induced *in vitro* by nutrient limitation-oxygen depletion (Trevijano-Contador et al., 2018), addition of polar lipids like PC (Chrisman et al., 2011), serum (Dambuza et al., 2018, Hommel et al., 2018), bacterial components like MTP and quorum sensing molecules (Albuquerque et al., 2014; Hommel et al., 2018; Dambuza et al., 2018). Other signals that contribute titanization include interaction of Ste3a pheromone receptor with alpha pheromone (Okagaki et al., 2010) and interaction of G protein-coupled receptor Gpr5 with a still unknown ligand (Okagaki et al., 2011).

Titan cells fail to undergo phagocytosis due to their large size, which also reduces the phagocytosis of normally sized cryptococcal cells (Crabtree et al., 2012; Okagaki and Nielsen, 2012) (**Figure 2**). Additionally, titan cells may prevent clearance by host immune cells, allowing them to maintain infection latency (Okagaki and Nielsen, 2012). During murine cryptococcal infection, titan cells promote disease through enhanced persistence in the host. Titan cells also promote the prevalence of lung eosinophils to stimulate a Th2 mediated immune response (Okagaki and Nielsen, 2012). Titan cells have been described to have interactions with amoeba (Chrisman et al., 2011) and wax moths (García-Rodas et al., 2011), suggesting these cells are formed under selective host pressures. Another interesting study demonstrated IgM inhibited titanization by inducing small capsules and downregulating the expression of stress and cell morphogenesis regulators (*RIM101*, *HOG1*), chitin synthetase (*CHS1*, *CHS2*, and *CHS8*) and cell wall carbohydrate synthetases (*AGS1* and *FKS1)* (Trevijano-Contador et al., 2020). Bioengineered cytotoxic T cells expressing GXM-targeting chimeric antigen receptor (GXMR-CAR) recognized and controlled titanization (Kumaresan et al., 2014). Furthermore, second generation GXMR-CAR redirected T cells to recognize variable polysaccharide thicknesses of *C. Gattii* and *C.neoformans*, reduced titanization and provided protection against pulmonary cryptococcosis (da Silva et al., 2021). Taken together, targeting titanization will likely have a profound impact on disease progression and prevention.

### Non-Lytic Exocytosis

*C. neoformans* has developed a fascinating mechanism, non-lytic exocytosis (or vomocytosis), to enhance its survival in hosts. This mechanism was first described by two groups independently, during imaging of macrophages infected with *C. neoformans* (Alvarez and Casadevall, 2006; Ma et al., 2006). Non-lytic exocytosis is the mechanism by which the live organisms are expelled out from a phagocyte, leaving both the host cell and the pathogen undamaged (**Figure 2**). Mammalian cells use non-lytic phagocytosis for a variety of functions, including phagocytosis, exocytosis of intracellular compartments required for phagosome formation, secretion of lysosomal enzymes and cytokines (Cruz-Acuña et al., 2019). During fungal infections, macrophages are the first responders. Macrophages recognize fungal cells based on their PAMPs and engulf them into phagosomes. Phagosomes then fuse with the lysosomes to form phagolysosomes where the invading fungi are killed (**Figure 2**). However, intracellular pathogens like *C. neoformans* and *C. gattii* have developed a novel mechanism to fuse their containment vacuoles with the cell membrane to evade lysosomal degradation (Alvarez and Casadevall, 2006; Ma et al., 2006; Bielska and May, 2016). Intracellular survival of *C. neoformans* inside the phagosome enables the ability to withstand oxidative burst, inflict damage to the phagolysosomal membrane, and impair critical host cell functions (De Leon-Rodriguez et al., 2018a). Interaction of *C. neoformans* and macrophages therefore would result either in 1) killing or restriction of *C. neoformans* growth by macrophages; 2) *C. neoformans* release following macrophage lysis; or 3) non-lytic exocytosis that results in the survival of both the macrophage and the fungus. Furthermore, non-lytic exocytosis could also be accompanied by a Trojan horse mechanism, whereby the fungus hijacks macrophages to disseminate into the brain to cause fatal cryptococcal meningitis (De Leon-Rodriguez et al., 2018b) (**Figure 2**). Within the lungs, *C. neoformans* can also become latent and localize within giant cells or macrophages in the form of granulomas. This latent infection can reactivate under conditions of weakened immunity, resulting in intracellular replication and dissemination (Fu et al., 2018) (**Figure 2**). Therefore, the coexistence of protective and deleterious roles of macrophages in the progression of cryptococcosis warrants further investigation.

Early observations showed that non-lytic exocytosis does not occur with heat-killed *Cryptococcus* or with latex beads, suggesting that host cells have a mechanism to detect phagosomal cargo viability (Alvarez and Casadevall, 2006; Ma et al., 2006). Cryptococcal virulence factors like phospholipase B, urease, and capsule influence non-lytic exocytosis (Cox et al., 2000; Cox et al., 2001). Cryptococcal strains with defective phospholipase B, urease production or acapsular strains show decreased non-lytic exocytosis (Alvarez and Casadevall, 2006). Furthermore, non-acidified phagosome also supports vomocytosis. The link between phagolysosomal pH and non-lytic exocytosis can be further explained by the inability of acapsular and urease lacking strains to buffer the acidic pH resulting lower vomocytosis (De Leon-Rodriguez et al., 2018a; Fu et al., 2018).

Host cytoskeleton rearrangement is an obvious target for regulating expulsion of pathogens. Addition of cytochalasin D, inhibitor of actin polymerization is known to enhance vomocytosis of *C. neoformans* (Johnston and May, 2010). Furthermore, Johnston et al. demonstrated a new mechanism by which the host cell attempts to retain internalized cargo and inhibits vomocytosis *via* assembly of transient actin cages surrounding the phagolysosome (Johnston and May, 2010). For proper non-lytic exocytosis, the internal vesicle must be positioned near the edge of the cell. This positioning allows the vesicle and plasma membrane to fuse together and allow expelling of the cargo (Stukes et al., 2016). This function of bringing the membranes together is carried out by Annexin A2. Loss of Annexin A2 reduces phagocytosis and vomocytosis, both *in vitro* and *in vivo*, resulting in enlarged capsule. These findings suggest a capsule either too small or too large may also abrogate non-lytic exocytosis (Stukes et al., 2016). Other host factors influencing non-lytic exocytosis include host membrane integrity (Tucker and Casadevall, 2002), composition (Nolan et al., 2017), and autophagy (Nicola et al., 2012).

Macrophages are highly plastic in nature and respond differently to different environmental cues. M2 or alternatively activated macrophages show an increased intracellular proliferation rate of cryptococcal cells and reduced non-lytic exocytosis. Conversely, M1 activated macrophages show reduced intracellular proliferation but increased events of non-lytic exocytosis (Seoane et al., 2020). A plausible explanation for this might be that alternatively activated macrophages have increased iron levels to support fungal growth (Weiss et al., 1997), whereas in classically activated macrophages, cryptococcal cells attempt to escape immune attack by non-lytic exocytosis. Thus, the polarization status of macrophages also affects non-lytic exocytosis. Lastly, Gilbert et al. demonstrated the role of host mitogen-activated kinase ERK5 in the regulation of non-lytic exocytosis (Gilbert et al., 2017). Suppressing ERK5 with a chemical inhibitor resulted in a significant increase of non-lytic exocytosis (Gilbert et al., 2017). Overall, different biological and immune cues influence the rate of non-lytic exocytosis. Understanding non-lytic exocytosis could help in the design of novel strategies to inhibit vomocytosis and prevent dissemination.

### Other Fungal Proteins Required for *C. neoformans*-Macrophage Interaction

As a facultative intracellular pathogen, a key characteristic of *C. neoformans* is its ability to survive and replicate within the phagolysosome of macrophages (Huang et al., 2016). Multiple fungal proteins have been found to play a role in *C. neoformans*-macrophage interaction.

In cryptococcal infected macrophages, urease promotes yeast fitness by delaying intracellular replication, and reduces damage to the phagolysosomal membrane thus, enhancing fungal dissemination through non-lytic exocytosis (Fu et al., 2018). During infection, urease alkalizes and damages the surrounding cellular environment through the production of ammonia. In a murine model of cryptococcosis, urease aids the transcytosis of Blood Brain Barrier (BBB) (Olszewski et al., 2004) and also influences immune responses in the lungs (Osterholzer et al., 2009). A recent study also highlighted the role of urease in the growth and metabolism of *C. neoformans.* Urease positive strains show increased activity under nutrient-limited conditions at 37°C, whereas urease deficient strains showed higher melanin levels at 26°C, suggesting urease is closely linked to the functions of key metabolic pathways (Toplis et al., 2020). Moreover, phospholipase hydrolyzes phospholipids. Since Phospholipase B1 (Plb1) is active at 37°C and is stable in an acidic environment, Plb1 likely promotes *C. neoformans* proliferation and survival within macrophages. Indeed, Δ*plb1* mutants display growth and proliferation defects within macrophages, causing attenuated virulence by having reduced fungal burden in the lungs and reduced dissemination to the CNS (Santangelo et al., 2004; Maruvada et al., 2012). Overall, urease helps *C. neoformans* to both persistently enter and exit macrophages while phospholipase facilitates growth within macrophages.

Anti-phagocytic protein 1 (App1) is a cryptococcal protein that is secreted extracellularly and inhibits phagocytosis through a complement-mediated mechanism where App1 binds to complement receptor 3 and 2 (Stano et al., 2009). This function of App1 inhibits both binding and ingestion of yeast cells by macrophages (Luberto et al., 2003). App1 as a virulence factor is differentially expressed based on location. In pulmonary cryptococcosis, App1 is highly expressed in bronchioalveolar fluid (BAL) while weakly expressed in blood (Williams and Del Poeta, 2011). Similarly, expression of App1 is also upregulated in other body fluids like serum and cerebrospinal fluid (CSF) (Williams and Del Poeta, 2011). Overall, low glucose conditions stimulate increased App1 transcription and mRNA stability (Williams and Del Poeta, 2011).

Our recent studies revealed the potential function of fungal phospholipid membrane distribution in *C. neoformans* pathogenesis, including macrophage recognition during early pulmonary infection. These studies showed that expression of the regulatory subunit of lipid translocase (flippase) Cdc50 was highly induced when *C. neoformans* was co-cultured with the J774.16 macrophage cell line (Huang et al., 2016). Mutation of Cdc50 reduced phosphatidylserine (PS) inward translocation, leading to accumulation of exocytoplasmic PS (Huang et al., 2016). The *cdc50*Δ strain was engulfed and killed more efficiently than the wild-type strain, suggesting loss of fungal CDC50 leads to increased phagocytosis of *C. neoformans*. Furthermore, the *cdc50*Δ mutant was hypersensitive to macrophage-mediated killing, suggesting Cdc50 is essential for fungal survival in macrophages. Therefore, Cdc50 activity is likely required for counteracting the anti-microbial activities of host macrophages (Huang et al., 2016).

The hypersensitivity of the *cdc50*Δ mutant to macrophage killing may be due to not only its defect in membrane integrity, but also increased PS on the cell surface. Increased PS exposure on mammalian cell surfaces is known to act as a signal for macrophage recognition and phagocytosis. This is a classical feature of apoptotic cells for signaling their clearance by macrophages (Shor et al., 2016). It remains unclear how increased PS exposure on the fungal plasma membrane generates a signal that is recognized by host macrophages despite the presence of the capsule and thick cell wall. It is possible that macrophages may secrete factors that survey the environment and help recognize potential target cells for phagocytosis. Therefore, PS exposure on the cell surface may have multiple effects on *C. neoformans*-host interaction during infection, an intriguing future research direction.

Data from cryptococcus-macrophage interaction assays suggests fungal F-box protein (Fbp1) is required for fungal proliferation inside macrophages. The *fbp1*Δ mutant showed a defect in intracellular proliferation following phagocytosis, which likely contributes to its attenuated virulence. Fbp1 is part of the ubiquitin-proteasome system. The inositol phosphosphingolipid-phospholipase C1 (Isc1) substrate is required for fungal survival inside macrophage cells. This is consistent with the role of Fbp1 in regulating cryptococcus-macrophage interaction and fungal virulence. These findings suggest the Fbp1-mediated ubiquitin-proteasome pathway controls *C. neoformans* virulence by regulating fungal intracellular growth in macrophages. This findings also reveal a new determinant of fungal virulence that is mediated by post-translational regulation of inositol sphingolipid biosynthesis (Liu and Xue, 2014).

## Research Tools for Studying *C. neoformans*-Macrophage Interaction

Effective research methods and tools have been developed to observe and measure the complex interactions between *C. neoformans* and macrophages. These include primary cells and cell lines as well as imaging techniques, omics, and host systems such as knockout mouse models, clinical samples, and strains.

Visualizing the intracellular residence of *C. neoformans* in macrophages has greatly aided in understanding this intricate relationship. With the help of electron microscopy, researchers can observe the formation of large phagolysosomes and how *C. neoformans* makes it permeable to escape (Tucker and Casadevall, 2002). Real-time imaging and GFP-labelled *C. neoformans* strains have also played a key role in elucidating the complex interaction between macrophages and *C. neoformans*. This labelling approach has aided in the understanding of: (i) macrophage lysis (Ben-Abdallah et al., 2012), (ii) lateral transfer or non-lytic exocytosis of yeast cells (Alvarez and Casadevall, 2006; Ma et al., 2006; Alvarez and Casadevall, 2007; Ma et al., 2007), and (iii) fusion and division of macrophages (Luo et al., 2008) or successful phagocytosis. Using transmission electron microscopy, Nolan et al. illustrated the role of lipids in *C. neoformans*-macrophages interactions and how lipids contributes to pathogenesis (Nolan et al., 2017). Additionally, Nicola et al. described non-lytic exocytosis using flow cytometry of *in vitro* and *in vivo* in murine infection models (Nicola et al., 2011). Another study used 54 clinical isolates to compare clinical outcomes of patients with interaction of phagocytic cells *in vitro* using flow cytometry. This group observed clinical outcomes such as clearance of fungal burden, dissemination to the CNS or patient death strongly correlated with their *in vitro* studies (Alanio et al., 2011). Flow cytometry analyses can analyze large populations of cells in multiple parallel experimental conditions. Additionally, imaging flow cytometry visualizes and quantifies some interactions more efficiently.

Another factor that plays a key role in *C. neoformans*-macrophage interaction is the capsule. Using phase contrast microscopy, researchers can visualize the capsule of *C. neoformans* using India ink. However, it remains difficult to study the structure of capsule as the capsule is primarily composed of water (Maxson et al., 2007). Considering the fragile structure of the capsule, non-destructive methods like optical tweezers (de S Araujo et al., 2019), dynamic light scattering (DLS) (Wolf et al., 2014) and fluorescence microscopy with capsule binding antibodies can be used (Casadevall et al., 2019). Overall, visualization of *C. neoformans*-macrophage interaction has enumerated different mechanisms used by *C. neoformans* for disease progression.

While it is difficult to obtain human alveolar macrophages, it is easier to obtain peripheral blood monocytes (PBMCs) and differentiate them into macrophages *in vitro*. PBMC derived macrophages can provide an approximation of host responses to cryptococcal challenge. These cells also demonstrates how cytokines, chemokines and opsonin influence the phagocytotic capacity of macrophages (Nelson et al., 2020). Smith et al. showed that cryptococcus infected human PBMC derived macrophages prevented complete maturation of phagosomes, thus, rendering the environment favorable for cryptococcal replication (Smith et al., 2015). Using multi-omics approach (lipidomics, proteomics, and metabolomics), extracellular vesicles (EVs) from infected macrophages show unique protein and lipid signatures of activation, which serve as inter-macrophage communication to resist cryptococcal infection at distant sites (Zhang et al., 2021). Transcriptomic analysis shows these vesicles activate p53 immune related pathways in naïve macrophages, suggesting EVs prime naïve macrophages to a proinflammatory phenotype for fungicidal activity (Zhang et al., 2021).

Many studies have demonstrated the role of murine macrophages in cryptococcosis. Feldmesser et al. showed murine alveolar macrophages that harbor *C. neoformans* undergo cell damage, suggesting alveolar macrophages are indeed the first line of defense in pulmonary cryptococcosis (Kozel et al., 2000). Another study highlighted how *C. neoformans* modulates alveolar polarization following infection with high and low uptake clinical *C. neoformans* isolates. High uptake clinical isolates show increases in M2 associated genes, whereas low uptake isolates show increases in M1 associated genes (Hansakon et al., 2019). Taken together, *in vivo* mouse models and cell lines have further emphasized the role of M1 macrophages and Th1 cytokines for protecting hosts.

Alanio et al. designed new assays using flow cytometry, microscopy, and gene analysis to characterize the dormancy of several *C. neoformans* populations *in vitro* and *in vivo*. Their study showed that dormant cells persist *in vitro* and *in vivo* by maintaining low metabolic activity and delayed growth (Alanio et al., 2015). Another interesting model for studying cryptococcal latency is rats, as rat macrophages are resistant to *C. neoformans* infection (Shao et al., 2005). Although the rat immune system contains *C. neoformans* through granulomatous infection (Shao et al., 2005), the majority of the fungal cells are found inside epithelial cells and macrophages (Goldman et al., 2000). Furthermore, corticosteroid induced reactivation results in increased extracellular fungal cells (Goldman et al., 2000). Therefore, rats may serve as a useful system for modeling latency and reactivation in humans. In addition to murine infection models, unconventional hosts such as insects and amoeba also help in understanding intracellular replication mechanisms of *C. neoformans*. Furthermore, some of these unconventional hosts such as *Drosophila melanogaster*, and *Acathaamoeba castellanii* offer several unique advantages, such as low cost, large sample sizes and none of the bioethical concerns of mammalian animal hosts. (Steenbergen et al., 2001; Steenbergen et al., 2003; Qin et al., 2011).

## Future Perspective

As the first line of the innate host immune response against pathogens such as *C. neoformans*, understanding how macrophages develop, function, and interact with fungi is critical for infection control and in future development of immunotherapies. Identifying fungal specific factors for interaction with host macrophages will be critical in these endeavors. Dissecting the steps that are involved in the establishment of intracellular parasitism of *C. neoformans* and its intricate relationships with the host immune system would also help advance our understanding of the pathogenesis of cryptococcosis. Using the above-mentioned approaches, significant advances have been made towards understanding *C. neoformans*-macrophage interactions. However, multiple questions remain to be answered, these include: which receptors are involved in recognition of *Cryptococcus* spores and yeast cells? What protective immune signaling are activated? Which specific virulence traits directly modulate host immune responses? What molecular mechanisms are associated with *C. neoformans*-macrophage interactions? Another intriguing topic for future study is the potential long-term impact of *Cryptococcus*-macrophage interactions to the subsequent response of macrophages. The interactions of host macrophages with the fungi may contribute to the development of innate immune memory, which can either lead to a suppressed immune response (tolerance) or an enhanced immune response (trained innate immunity). Future studies may also reveal that fungal infection promotes trained immunity in epithelial cells and can enhance barrier immunity and anti-cryptococcal functions.

## Author Contributions

YW, SP, and KW contributed to writing and editing of the manuscript. OD, AR, and CX contributed to editing and revision of manuscript. All authors contributed to the article and approved the submitted version.

## Funding

This work is supported by a NIH grant R01AI141368 to AR and CX. Studies in the Xue lab are also supported by NIH grant R01AI123315, R21AI154318 and the Rutgers HealthAdvance Fund. AR holds an Investigators in the Pathogenesis of Infectious Disease Award from the Burroughs Wellcome Fund. YW was supported by a scholarship from Chinese Scholarship Council. The funders had no role in study design, data collection and interpretation, or the decision to submit the work for publication.

## Conflict of Interest

The authors declare that the research was conducted in the absence of any commercial or financial relationships that could be construed as a potential conflict of interest.

## Publisher’s Note

All claims expressed in this article are solely those of the authors and do not necessarily represent those of their affiliated organizations, or those of the publisher, the editors and the reviewers. Any product that may be evaluated in this article, or claim that may be made by its manufacturer, is not guaranteed or endorsed by the publisher.
